# Insulin-like growth factor-1/insulin-like growth factor-1 receptor signalling in macrophages facilitates recovery from acute lung injury

**DOI:** 10.1080/07853890.2025.2606565

**Published:** 2025-12-22

**Authors:** Miyuki Niisato, Masahiro Yamashita, Yasushi Kawasaki, Hiroshi Furukawa, Takashi Sato, Ichiro Kawada

**Affiliations:** ^a^ Department of Pulmonary Medicine, Iwate Medical University School of Medicine, Shiwa, Japan; ^b^ Department of Pharmaceutical Health Science, Faculty of Pharmacy, Iryo Sosei University, Japan; ^c^ Department of Rheumatology, NHO Tokyo National Hospital, Kiyose, Japan; ^d^ Technical Support Center for Life Science Research, Iwate Medical University School of Medicine, Shiwa, Japan

**Keywords:** Macrophages, Insulin-like growth factor-1, acute lung injury, efferocytosis, acute respiratory distress syndrome

## Abstract

**Background:**

No effective treatment strategy for acute respiratory distress syndrome (ARDS) has been established. Conflicting reports on the effects of insulin-like growth factor (IGF)-1 stimulation and the inhibition of IGF-1 receptor (IGF-1R) signalling in tissue injury across several organs have led to hesitation in advancing IGF-1-based treatment strategies for tissue damage.

**Objective:**

We aim to examine whether IGF-1/IGF-1R signalling contributes to recovery from acute lung injury in mouse models and to further explore its potential mechanisms.

**Methods:**

Lipopolysaccharide (LPS) was intratracheally injected into mice to create acute lung injury models. Experiments were conducted to acquire or inhibit IGF-1 signalling through the intratracheal injection of recombinant IGF-1 or JB1, an IGF-1 receptor antagonist during the recovery phase of the models, starting on day 4 after LPS administration. Bone marrow monocyte-derived macrophages (MDMs) cocultured with IGF-1 and/or JB1 were intratracheally injected during the recovery phase.

**Results:**

Inflammatory cell counts and lung injury scores were significantly decreased when recombinant IGF-1 was administered in the later phase, while they increased with the administration of JB1. On day 4 after LPS injection, IGF-1 receptor (IGF-1R, also known as CD221) was strongly expressed on macrophages, particularly in CD11c^+^SiglecF^+^ alveolar macrophages (AMs). Intratracheal injection of MDMs cocultured with IGF-1 decreased lung neutrophil counts, whereas the addition of JB1 to MDMs cocultured with IGF-1 counteracted the effect of IGF-1. JB1 also reduced efferocytosis capacity of AMs in the later phase. In the phagocytosis assay, LPS decreased the efflux capacity of macrophages, but recombinant IGF-1 improved this capacity regardless of the presence or absence of LPS.

**Conclusion:**

IGF-1/IGF-1R signalling in macrophage facilitates the recovery from acute lung injury *via* enhancing efferocytosis. IGF-1 delivery potentially offers a new treatment strategy for ARDS.

## Introduction

Acute respiratory distress syndrome (ARDS) occurs as a result of infections, trauma, drugs and connective tissue diseases [1,2]. Very limited therapeutic strategies are available for this condition, and its prognosis remains unsatisfactory. Severe ARDS may require prolonged ventilation and is associated with a high mortality rate [3]. ARDS represents a pathologic pattern of diffuse alveolar damage [4]. During the later phase, macrophages remove apoptotic neutrophils from the site of inflammation, a process known as efferocytosis, which is crucial for effective resolution [5,6]. In recent years, specimens collected from ARDS patients have provided increasing information on the pathogenic role of impaired efferocytosis in macrophages in ARDS [7–9].

Insulin-like growth factor (IGF)-1 signalling is known to play important roles in the development, metabolism, and survival of many cell types and organs. IGF-1 shows high binding affinity to the IGF-1 receptor (IGF-1R), but also has a weak binding ability for insulin receptors [10]. Over the past two decades, there have been conflicting reports on the effects of IGF-1 stimulation and the inhibition of IGF-1R signalling on the acute onset of tissue injury in several organs [11–21]. Some studies have indicated that IGF-1 stimulation alleviated tissue injury, while the inhibition of IGF-1R signalling also reduced acute injury. However, these reports have primarily focused on the acute phase of tissue injury, and have been limited to examining either IGF-1 stimulation or IGF-1R signalling inhibition. Conversely, the role of IGF-1 signalling in the recovery phase remains not fully understood. In the present study, we found that IGF-1 signalling facilitates recovery from acute lung injury Together with the previous reports, our findings consistently demonstrated that IGF-1 has positive effects, while inhibition of IGF-1R signalling consistently delayed the recovery. This indicates that the inhibition of IGF-1R signalling has different actions on lung injury in a phase-dependent manner. Therefore, IGF-1 delivery may offer a potential treatment strategy for ARDS in humans.

## Materials and methods

### Mice

We acquired wild-type C57BL/6J mice from NIHON SLC in Tokyo, Japan, and macrophage Fas-induced apoptosis (MaFIA) mice from the Jackson Laboratory located in Sacramento, California, USA. In this experiment, MaFIA mice were used as reporters to detect monocytic cells, which express enhanced green fluorescent protein (EGFP) exclusively in cells expressing macrophage colony-stimulating factor receptors (M-CSFR). These transgenic mice were bred onto the C57BL/6J background. The experimental protocols for using C57BL/6J mice and MaFIA mice were approved by the Committee for Animal Experiments of Iwate Medical University (29-013, 30-016, 05-022). The research institution is from March 29, 2017, to March 31, 2026.

### LPS-ALI model

In the mouse model using LPS (Sigma-Aldrich, St. Louis, MO, USA, from Escherichia coli O111), it was dissolved in sterile saline (100 μL) and administered intranasally to randomly selected 8–12 week-old female mice anaesthetized with sevoflurane. The experimental unit is a single animal. The sample size was chosen to be the minimum number necessary to achieve statistical significance. We used 1.9 mg/kg body weight of LPS as acute lung injury models through the present study, and harvested the mice at each time points. The mice were randomly assigned to groups, and the first author confirmed the group assignments and the evaluation of results. We intratracheally administered 1 μg per body of recombinant IGF-1 (R&D systems, Minneapolis, MN, USA) or 20 μg per body of JB1 (Sigma-Aldrich) on days 3 and 4 after LPS injection, with a vehicle control group included, followed by harvest on day 5. These compounds were diluted in 100 μL of sterile saline prior to administration after inducing anaesthesia with sevoflurane. We adopted a reduction of 20% or more in body weight as the humane endpoint, and euthanasia would be performed by cervical dislocation in accordance with these humane endpoints if necessary. On day 5 of harvesting, there were no mice that met the criteria for the humoral endpoint. Five days after LPS was administered to mice, ultraviolet (UV)-irradiated PKH-labelled exogenous apoptotic neutrophils (3 × 10^6^ per mouse in 100 μL phosphate-buffered saline [PBS]) were injected into the airway. Bronchoalveolar lavage (BAL) samples were collected 30 min later. Experimental units were evaluated at the individual level. There was no exclusion for animals (or experimental units) under experimentation and data points under analysis. Potential confounding factors in breeding and experimentation are not taken into account. Confounders were not controlled.

### BAL collection

Mice were euthanized at each required time point and BAL cells and BAL fluid were collected. After euthanasia, the lungs were perfused with 5 mL of PBS from the right ventricle. Bronchial lavage was performed using 1.0 mL of PBS on two occasions. The resulting cells were counted using a cell counter. Trypan blue staining was used to exclude dead cells from analysis. Cell-free BAL fluid was stored in a −80 °C freezer for cytokine measurements. The primary outcome measure used to determine the sample size was the number of BAL neutrophils.

### Bone marrow MDMs

Mononuclear cells were isolated from bone marrow using a histopaque density gradient centrifugation method with histopaque 1083^®^ (Sigma-Aldrich, St. Louis, Mo). The isolated mononuclear cells were resuspended in Poly D-Lysine-coated dishes for attachment. The adherent cells were cultured with RPMI supplemented with 10% foetal calf serum, 100 U/mL penicillin, and 100 μg/mL streptomycin. In addition, 60 ng/mL of recombinant mouse macrophage colony-stimulating factor (M-CSF) (Biolegend, San Diego, CA, USA) was added to the bone marrow mononuclear cells to obtain bone marrow monocyte-derived macrophages (MDMs).

### ELISA

We estimated BAL fluid levels of pro-inflammatory cytokines responsive to inflammation, including myeloperoxidase, C-X-C motif chemokine ligand (CXCL) 1, CXCL2, interleukin (IL)-1beta, tumour necrosis factor (TNF)-α, monocyte chemotactic protein (MCP)-1, and IGF-1 and transforming growth factor (TGF)-β, as well as anti-inflammatory cytokines including IL-10. Enzyme-linked immunosorbent assay (ELISA) kits obtained from eBioscience (Santa Clara, CA, USA) were used to evaluate mouse IL-10 levels. Duoset or Quantikine ELISA kits obtained from R&D Systems were used for murine cytokines other than IL-10 in accordance with the manufacturer’s instructions.

### Histopathology and cytochemistry

Mouse tissue samples were fixed in 10% formalin and embedded in paraffin. Thereafter, 4-mm-thick sections were cut and stained with haematoxylin and eosin. Histological lung injury was assessed according to previously described methods [8]. A quantified assessment of injury was conducted in a blinded manner by grading four histological findings: haemorrhage, inflammation, atelectasis, and oedema. Each feature was rated on a scale from 0 (normal) to 4 (diffuse abnormality). A composite average lung injury score was calculated for each mouse, with values ranging from 0 (indicating normal) to 16 (indicating maximum injury).

### Fluorescence-activated cell sorting (FACS)

Monocyte lineage cells were isolated using fluorescence-activated cell sorting (FACS). Initially, blood cells were treated with anti-mouse CD16/32 antibodies (BD Biosciences, Franklin Lakes, NJ, USA) to block Fc receptors, followed by staining with a combination of fluorochrome-conjugated antibodies. The cells were not subjected to permeabilization for intracellular staining. Primary antibodies targeting Ly6G (clone RB6-8C5), CD11c (clone H.K 1.4), CD11b (clone M1/70), Ly6C (clone HK1.4) and Siglec F (clone S17007L) were sourced from BioLegend Inc. (San Diego, California, USA). Primary antibody against CD221 (polyclonal) was purchased from Bioss (Woburn, MA, UAS). Data acquisition and analysis were conducted using a BD Canto II Flow Cytometer along with BD FACS Diva software (version 8; BD Biosciences). Dead cells were identified using 7-amino-actinomycin D (7-AAD) (BD Biosciences) staining.

### Generation of apoptotic neutrophils

To verify the effects of IGF-1 and JB1 on the efficiency of macrophage efferocytosis,　purified neutrophils were harvested from the spleens of the same animals injected with extrinsic neutrophils. Following the previous report, neutrophils were separated using a magnetic microbead-based antibody technique (autoMACS; Miltenyi Biotec, Bergisch, Germany) [8]. Neutrophils were labelled using the PKH26 Red Fluorescent Dye Linker Kit (Sigma-Aldrich) or pHrodo Red (Thermo Fisher Scientific) in accordance with the manufacturer’s instructions. Dye-labelled Neutrophils labelled with dye were subjected to UV light exposure for a duration of 15 min. These neutrophils were diluted in 100 μL of sterile saline prior to administration after inducing anaesthesia with sevoflurane.

### In vitro phagocytosis assay

MDMs were cultured at 1.0 × 10^5^ cells/well in four-chamber slides (chambers mounted on glass slides with covers; Eppendorf, Hamburg, Germany) for 1 h. Simultaneously, LPS (0.25 μg/mL), recombinant IGF-1 (0.1 μg/mL), JB1 (5.0 μg/mL), TAK242—a small-molecule-specific inhibitor of Toll-like receptor (TLR)-4 (ChemScene LLC, Monmouth Junction, NJ, USA, 2.0 μg/mL) or CD51/61 blocking antibody (23C6, 10 μg/mL), or isotype controls were added to the chamber slides. MDMs were incubated for 2 h at 37.0 °C in 5% CO_2_. Afterward, 5.0 × 10^5^ UV-irradiated pHrodo-labelled neutrophils, suspended in 100 µL of RPMI1640 supplemented with 10% foetal calf serum were seeded onto cultured macrophages. Washing twice in PBS removed non-ingested neutrophils from the chamber slide. Phagocytosis index was calculated by fluorescence microscopy as the number of MDMs phagocytosed by pHrodo^+^ cells divided by the total number of MDMs.

This study was conducted in accordance with the ARRIVE (Animal Research: Reporting of *In Vivo* Experiments) guidelines to ensure the transparent reporting of animal research.

### Statistical analysis

Each experiment was conducted twice. Data are expressed as mean ± standard error of the mean (SEM) values. Data are reported for the full cohort. The normality of distribution was estimated using the Kolmogorov–Smirnov test. Differences in measured variables between the experimental and control groups were assessed using Student’s *t*-tests. One-way or Two-way analysis of variance followed by Tukey’s or Dunnett’s post-hoc tests was used for multiple group comparisons in the parametric analyses. Statistical analyses were conducted with the IBM SPSS Statistics software (IBM Japan, Tokyo, Japan). Differences with a *p* value less than 0.05 were considered statistically significant.

## Results

### IGF-1 plays a role in the recovery phase during LPS-induced lung injury

**Figure 1.**
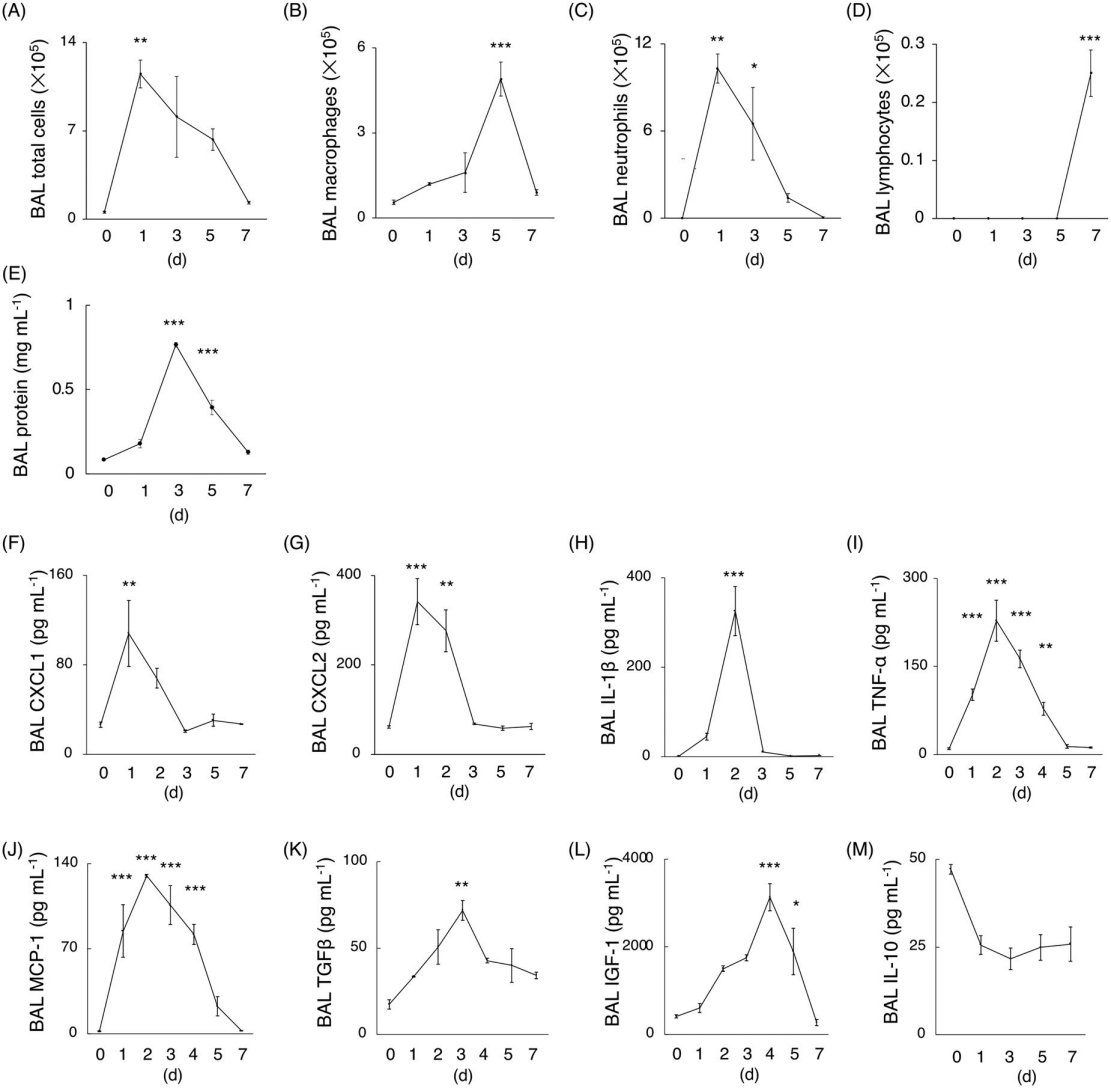
The kinetics of BAL immune cell counts and cytokine levels in a LPS-induced lung injury murine model. (A–M) The kinetics of bronchoalveolar lavage (BAL) immune cell counts and levels of various cytokines in 1.9 mg/kg body weight of lipopolysaccharide (LPS)-induced lung injury models were analyzed using an enzyme-linked immunosorbent assay (ELISA) (n = 4–6 mice per group). (A) total cell counts, (B) macrophage counts, (C) neutrophil counts, and (D) lymphocyte counts. (E) protein concentration. (F) C-X-C Motif Chemokine Ligand (CXCL) 1, (G) CXCL2, (H) interleukin (IL)-1β, (I) tumour necrosis factor (TNF)-α, (J) monocyte chemoattractant protein 1 (MCP-1), (K) transforming growth factor (TGF)-β, (L) insulin-like growth factor (IGF)-1 and (M) IL-10. Data are presented as line charts showing the mean ± standard error of the mean (SEM) values. Differences among multiple groups were analyzed using one-way ANOVA followed by Dunnett’s *post hoc* tests in comparison with the levels in day 0. *p < 0.05, **p < 0.01, ***p < 0.001.

We determined the kinetics of BAL immune cell counts and cytokine levels in LPS-induced lung injury models using C57BL/6J mice (Figures 1A–M). BAL total cell and neutrophil counts significantly increased on day 1 after LPS administration compared to day 0. In contrast, BAL macrophage and lymphocyte counts significantly increased on day 5 and 7, respectively. We categorized the early phases of acute lung injury as occurring before day 3, characterized by neutrophils as the principal inflammatory cells in the lung tissue, and the recovery phase as beginning after day 4, when neutrophilic inflammation in the lungs began to regress and macrophages became predominant. BAL levels of CXCL1, CXCL2, IL-1β, TNFα and TGFβ were increased during the early phase, while IGF-1 levels were significantly increased in the later phase, suggesting that IGF-1 is involved in the resolution processes of acute lung injury.

### The effects of IGF-1/IGF-1R signalling in the recovery from lung injury

**Figure 2.**
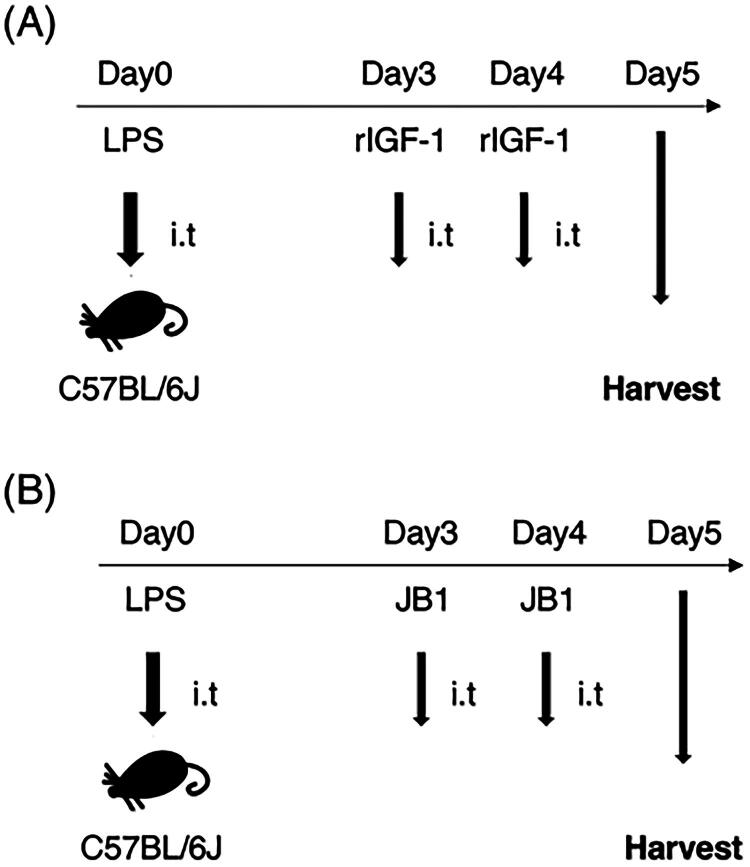
Experimental protocol for lipopolysaccharide (LPS)-induced lung injury mice. (A) Recombinant insulin-like growth factor-1 (IGF-1) was administered intratracheally during the recovery phase in the LPS-induced model at a dose of 1.9 mg/kg. (B) JB-1, an antagonist to IGF-1, was administered intratracheally during the recovery phase in the LPS-induced model at a dose of 1.9 mg/kg.

**Figure 3.**
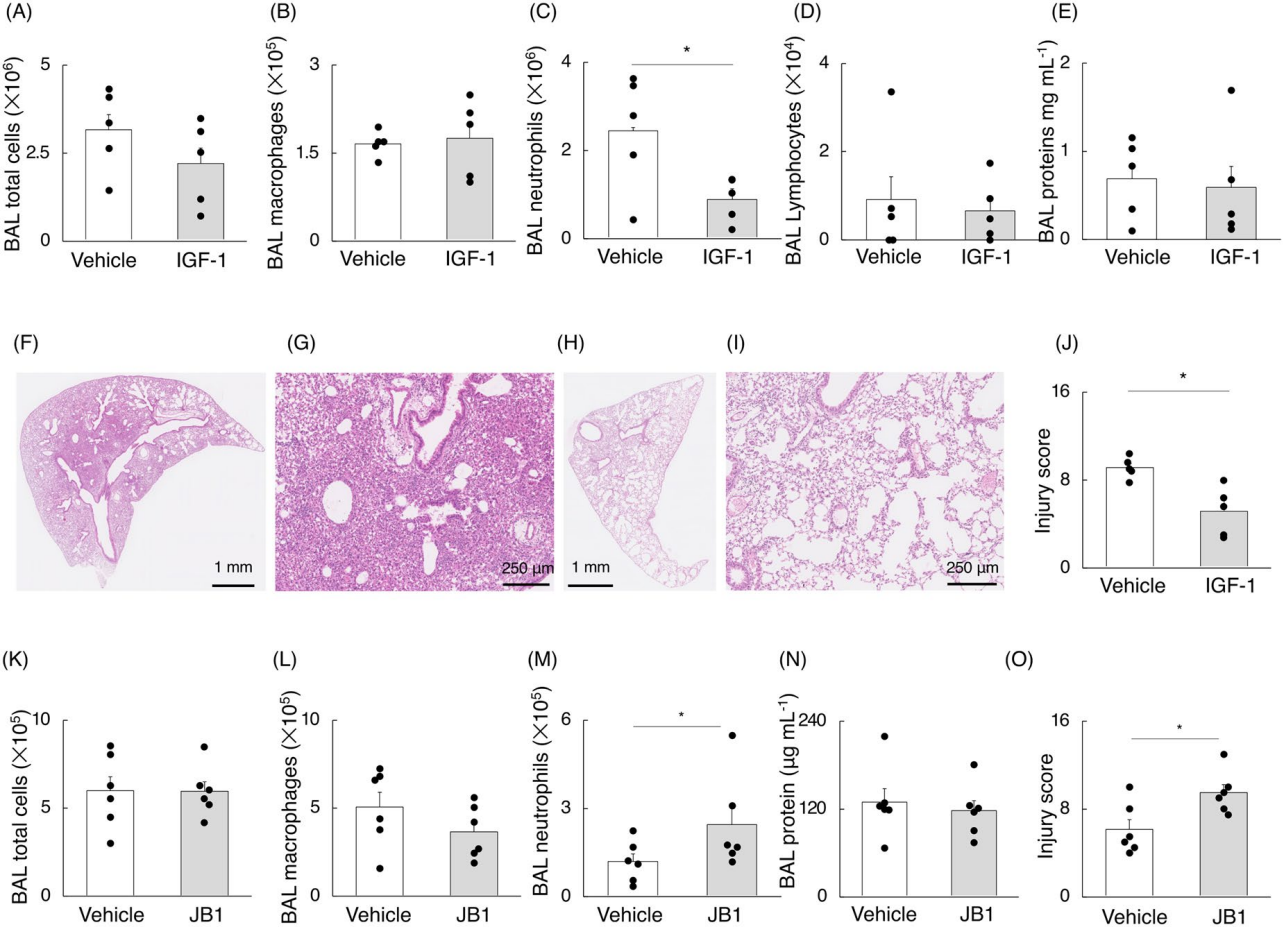
Recovery effects of IGF-1/IGF-1R signalling in the later phase. A dose of 1.9 mg of lipopolysaccharide (LPS) per kg of body weight was intranasally injected to create acute lung injury models. Insulin-like growth factor (IGF)-1 or vehicle was intranasally injected on days 3 and 4 after the administration of LPS, followed by harvest at day 5 (n = 5 mice per group). (A) Bronchoalveolar lavage (BAL) total cell counts, (B) macrophage counts, (C) neutrophil counts, (D) lymphocyte counts, and (E) BAL fluid protein concentration. (F–I) Representative H&E staining of lung tissue in mice receiving (F, G) vehicle and (H, I) recombinant IGF-1 at day 5 after LPS injection. (J) Injury score. (K-O) JB1—an antagonist peptide specific to the IGF-1 receptor or vehicle was intranasally injected at days 3 and 4 after the administration of LPS, followed by harvest at day 5 (n = 6 mice per group). (K) BAL total cell counts, (L) macrophage counts, (M) neutrophil counts, (N) BAL fluid protein concentration, and (O) injury score. The data are presented as bar graphs and dot plots showing the mean ± standard error of the mean (SEM). Differences between two groups were analyzed using Student’s *t*-tests. *p < 0.05.

We examined the effects of intratracheal administration of recombinant IGF-1 during the recovery phase in the LPS-induced models (Figure 2A and Figures 3A–J). BAL neutrophil counts were significantly lower in mice receiving recombinant IGF-1 on days 3 and 4 after LPS injection compared to those receiving vehicle (Figures 3C). BAL lymphocyte counts did not differ between the two groups and were extremely low compared to neutrophil and macrophage counts (Figure 3D). The injury score on day 5 after LPS injection was significantly lower in mice receiving recombinant IGF-1 compared to those receiving vehicle (Figures 3F-I). Next, we investigated whether the effects of IGF-1R signalling produced effects opposite to those of IGF-1 signalling. JB1, an antagonist specific to IGF-1R, was intratracheally injected on days 3 and 4 into lung injury models of C57BL/6J mice, and the mice were harvested the following day (Figure 2B, Figures 3K–O). Consistently, BAL neutrophil counts and injury scores were significantly higher in mice receiving JB1 compared to those in the vehicle group (Figure 3M). Therefore, IGF-1/IGF-1R signalling facilitates the recovery from acute lung injury.

### The effector cells of IGF-1/IGF-1R signalling in the recovery from lung injury

**Figure 4.**
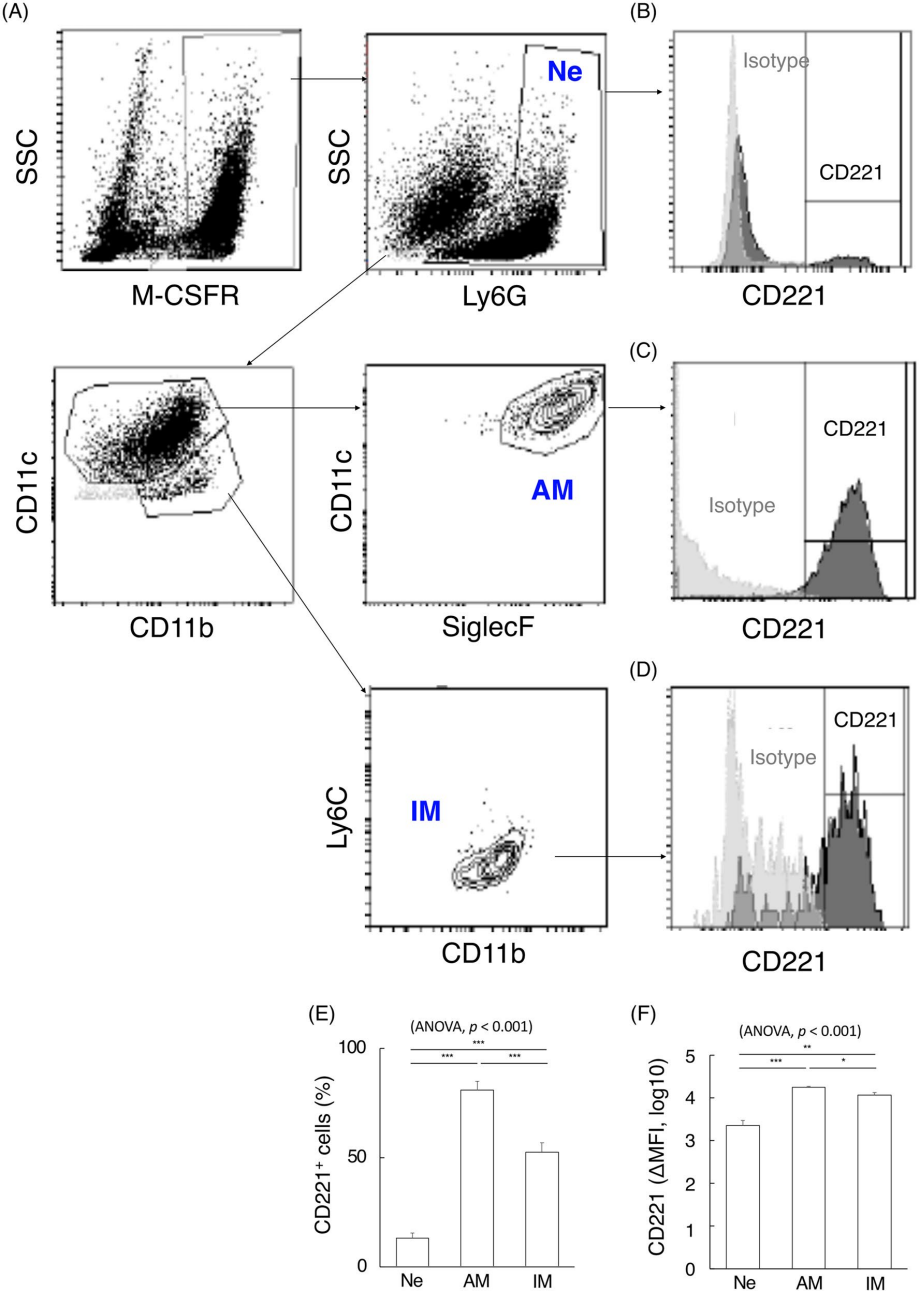
The effector cells of IGF-1/IGF-1R signalling in the recovery from lung injury. (A) The images present flow cytometry (FACS) diagram of bronchoalveolar lavage (BAL) neutrophils and monocyte lineage cells expanded by macrophage colony-stimulating factor receptor (M-CSFR), Ly6G, CD11c, CD11b, SiglecF and Ly6C. (B-D) CD221 expression in BAL neutrophils, alveolar macrophages, and interstitial macrophages. (E, F) Comparison of the rates of CD221-positive cells, and delta mean fluorescence intensity of CD221 among the three types of cells (n = 5 per group). The data are presented as bar graphs as the mean ± standard error of the mean values. Differences among multiple groups were analyzed using one-way ANOVA followed by Tukey’s *post hoc* tests. *p < 0.05, **p < 0.01, ***p < 0.001.

To explore effectors cells of IGF-1/IGF-1R signalling on the later phase, LPS was intratracheally injected into MaFIA mice, followed by harvest on day 4, and performed BAL. We examined the expression levels of IGF-1R (also known as CD221) on BAL neutrophils and monocyte lineage cells, which are likely directly related to decreased BAL neutrophil counts. Based on the results of FACS combined with May-Giemsa staining performed after cytospinning BAL cells, M-CSFR^+^Ly6G^+^ neutrophils and M-CSFR^+^Ly6G^-^ monocyte lineage cells accounted for 55.5 ± 6.3% and 44.3 ± 6.4% of total BAL immune cells, respectively (Figure 4A). M-CSFR^+^Ly6G^-^CD11c^+^CD11b^+^SiglecF^+^ alveolar macrophages (AMs) and M-CSFR^+^CD11c^-^CD11b^+^Ly6C^-^ interstitial macrophages (IM) represented 80.1 ± 2.3% and 10.1 ± 0.6% of BAL monocytic cells, respectively (Figure 4A). CD221-positive cells on day 4 in the LPS-induced lung injury model were 13.2 ± 2.3% in neutrophils, 80.9 ± 4.1% in AMs, and 52.6 ± 4.2% in IMs (Figures 4B-D). CD221 was significantly more strongly expressed on BAL AMs than on neutrophils or IMs, both in terms of positive cell counts and *Δ* Mean Fluorescence Intensity, while BAL neutrophils were weakly expressed than the two types of macrophages (Figures 4E, F). Thus, it was speculated that AM-dominated macrophages are the primary effector cells for late IGF-1R signalling.

### Involvement of macrophages in IGF-1/IGF-1R-mediated recovery effects

**Figure 5.**
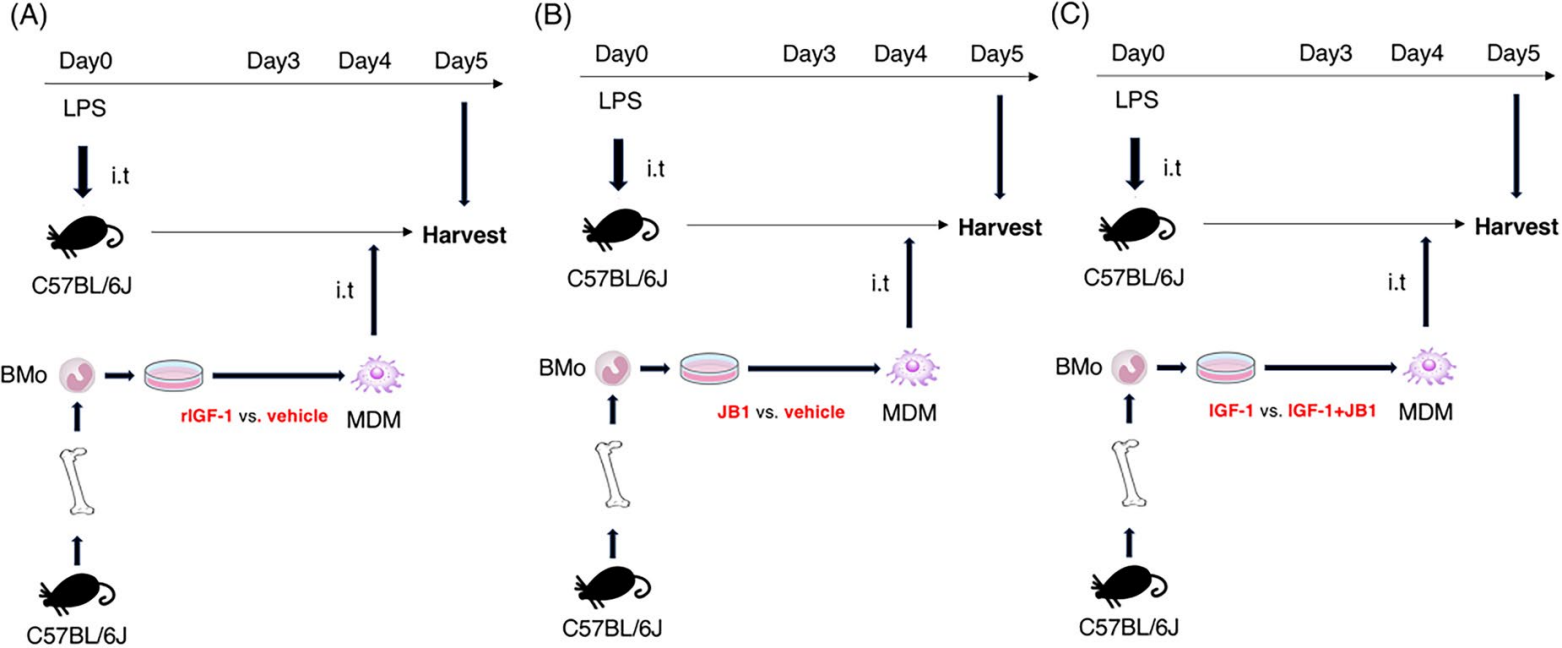
Airway administration protocol of bone marrow-derived macrophages (MDMs) during the recovery phase of LPS-induced lung injury. Bone marrow-derived macrophages (MDMs) obtained from C57BL/6J mice are generated, and cocultured with (A) insulin-like growth factor-1 (IGF-1) or vehicle, (B) JB1 or vehicle, and (C) IGF-1 or IGF-1 and JB-1 overnight. A dose of 1.9 mg of lipopolysaccharide (LPS) per kg of body weight was intranasally injected into other C57BL/6J mice. The MDM cells (1.0 × 10^6^) were intratracheally delivered to mice receiving LPS on day 4 after LPS injection, followed by harvest on day 5 after LPS injection.

**Figure 6.**
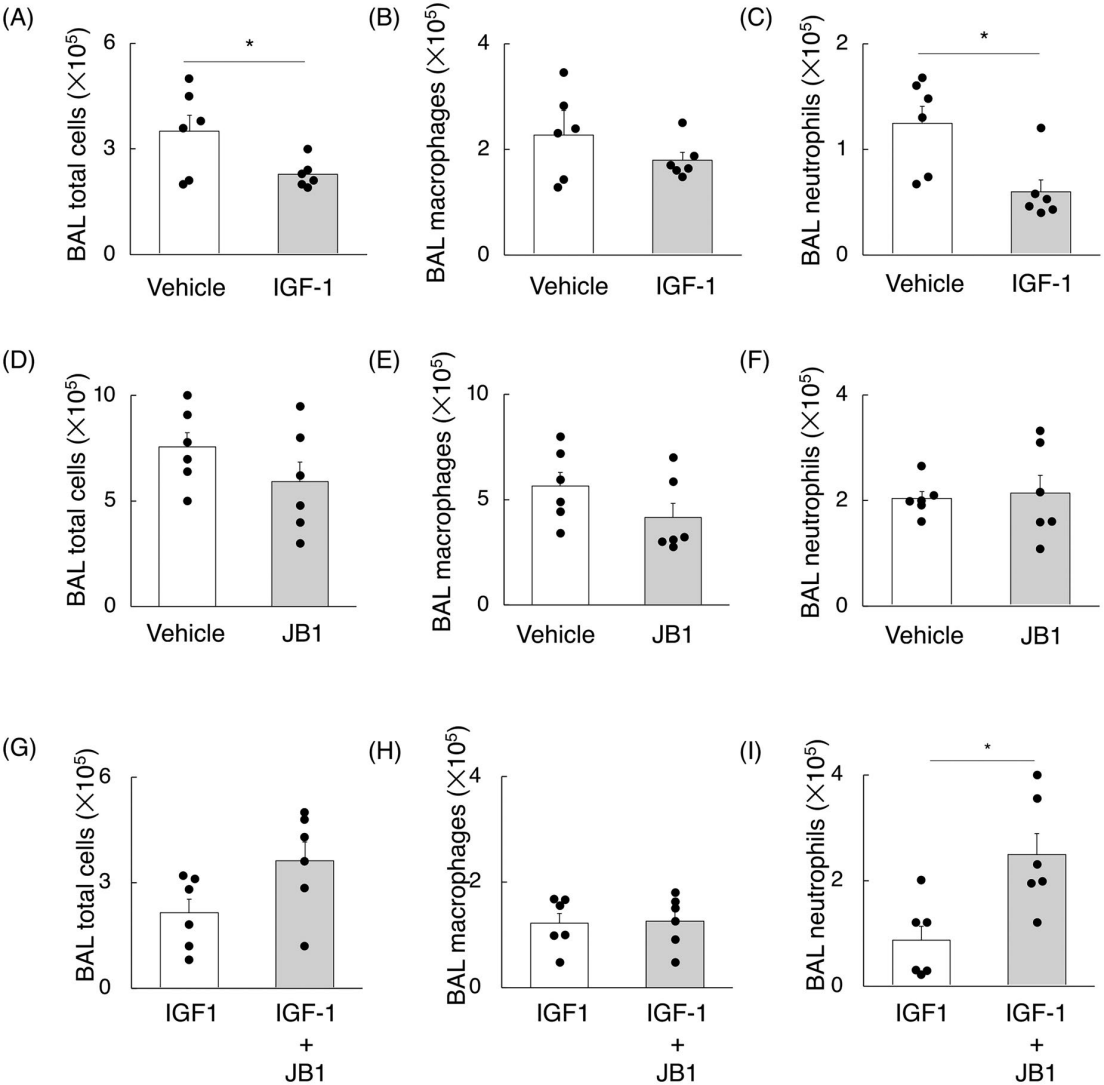
Impact of IGF-1/IGF-1R signalling on macrophages in BAL neutrophil reduction. (A-C) Bone marrow-derived macrophages (MDMs) obtained from C57BL/6J mice are generated, and cocultured with IGF-1 or vehicle overnight. A dose of 1.9 mg of Lipopolysaccharide (LPS) per kg of body weight was intranasally injected into other C57BL/6J mice. The MDM cells (1.0 × 10^6^) were intratracheally delivered to mice receiving LPS at day 4 after LPS injection, followed by harvest at day 5 after LPS injection (n = 6 mice per group). (A) Bronchoalveolar lavage (BAL) total cell counts, (B) macrophage counts, and (C) neutrophil counts. (D-F) MDMs obtained from other C57BL/6J mice were generated, and cocultured with JB1 or vehicle overnight. The MDM cells (1.0 × 10^6^) are intratracheally delivered into mice receiving LPS at day 4 after LPS injection, followed by harvest at day 5 after LPS injection (n = 6 mice per group). (D) BAL total cell, (E) macrophage, and (F) neutrophil counts. (G-I) MDMs obtained from other C57BL/6J mice are generated, and cocultured with IGF-1 and JB1, or IGF-1 and vehicle overnight. The MDM cells (1.0 × 10^6^) are intratracheally delivered into mice receiving LPS at day 4 after LPS injection, followed by harvest at day 5 after LPS injection (n = 6 mice per group). (G) BAL total cell, (H) macrophage, and (I) neutrophil counts. The data are presented as bar graphs and dot plots showing the mean ± standard error of the mean (SEM). Differences between two groups were analyzed using Student’s *t*-tests. *p < 0.05.

To determine whether macrophages are involved in the effect of recombinant IGF-1 in reducing BAL neutrophil numbers during the recovery phase, 1.0 × 10^6^ MDMs that were cocultured with IGF-1 or vehicle were intratracheally injected on day 4 after LPS administration, followed by harvesting the next day. BAL total cell and neutrophil counts significantly decreased in mice receiving MDMs cocultured with recombinant IGF-1 compared to vehicle control, while there was no statistical difference in BAL macrophage counts (Figure 5A and Figure 6A–C). Next, to determine the effect of IGF-1R-inhibited macrophages on recovery from acute lung injury, 1.0 × 10^6^ MDMs that were cocultured with JB1 or vehicle were intratracheally injected on day 4 after LPS administration, followed by harvesting the next day. There were no statistical differences in BAL cell counts between IGF-1R-inhibited MDMs and control MDMs (Figure 5B and Figure 6D–F). Furthermore, when MDMs cocultured with recombinant IGF-1 and JB-1 were injected intratracheally into mice on day 4 after LPS administration, BAL neutrophil counts significantly increased compared to mice receiving MDMs cocultured with IGF-1 and vehicle, suggesting that the recovery effects of IGF-1 were primarily mediated by IGF-1R signalling on macrophages (Figure 5C and Figures 6G-I).

### Involvement of efferocytosis in IGF-1/IGF-1R-mediated recovery effects

**Figure 7.**
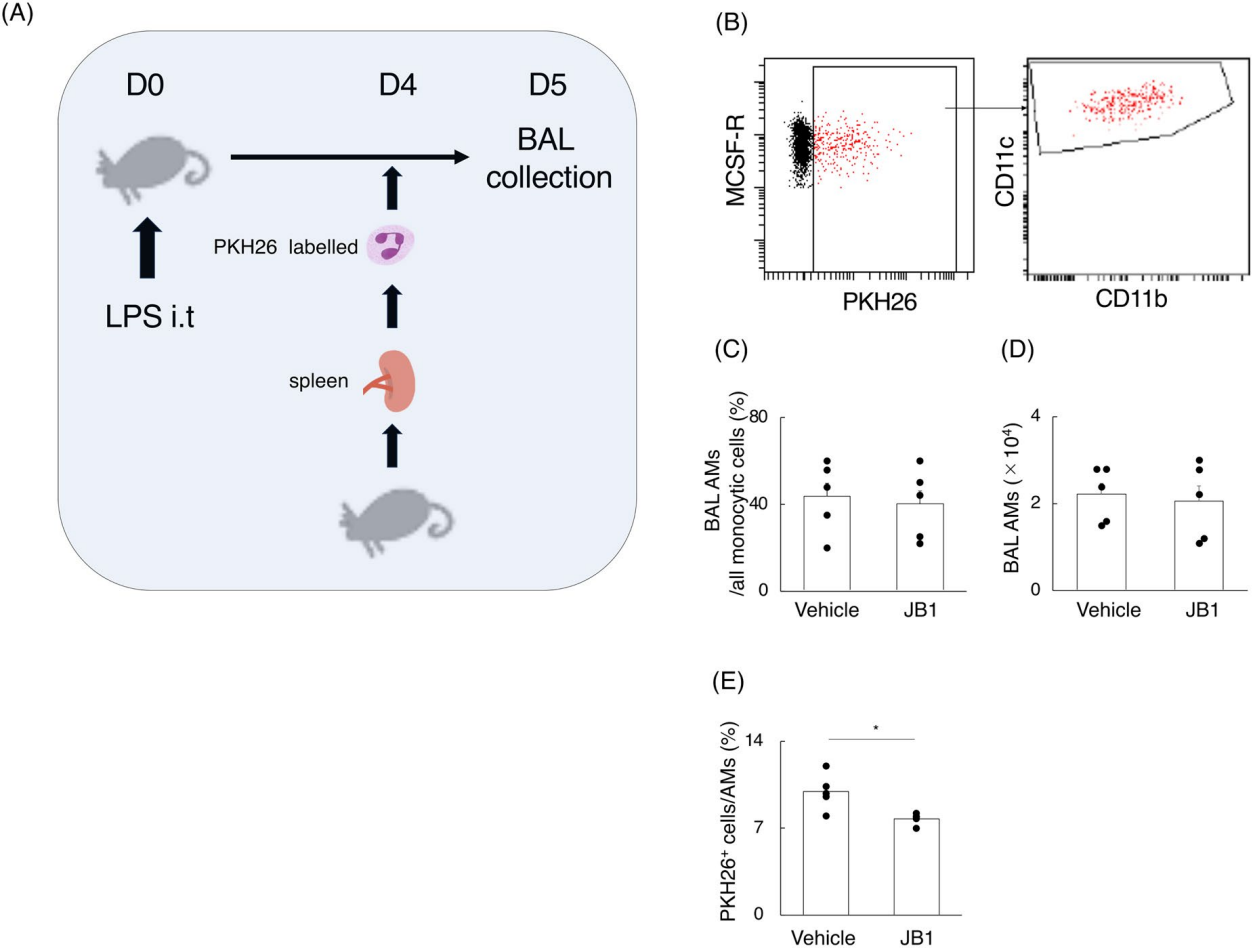
Inhibitory effects of the IGF-1R antagonist on macrophage efferocytosis during the later phase of acute lung injury. (A). Schema of the mouse model. A dose of 1.9 mg of Lipopolysaccharide (LPS) per kg of body weight was intranasally injected into C57BL/6J mice. Neutrophils obtained from spleens of other C57BL/6J mice were irradiated, followed by labelling with PKH26. PKH26-labelled neutrophils (3.0 × 10^6^) were intratracheally injected into C57BL/6J mice receiving LPS on day 5. C57BL/6J mice receiving LPS were harvested 1 hour after neutrophil injection, and alveolar cells were collected by bronchoalveolar lavage (BAL). (B) The pictures present flow cytometry (FACS) diagram of macrophage colony-stimulating factor receptor (M-CSFR)^+^Ly6G^-^PKH26^+^ monocytic cells expanded by CD11c and CD11b. (C) The rates of AMs among all monocyte lineage cells (n = 5 per group). (D) BAL AM counts. (E) The rates of PKH26^+^ cells phagocytosed by AMs. The data are presented as bar graphs and dot plots showing the mean ± standard error of the mean (SEM). Differences between two groups were analyzed using Student’s *t*-tests. *p < 0.05.

Next, we examined whether efferocytosis is involved in the IGF-1/IGF-1R-mediated recovery effects in macrophages. We generated UV-irradiated PKH26^+^ neutrophils collected from the spleen of MaFIA mice, and intratracheally injected 1.0 × 10^6^ neutrophils into other MaFIA mice that received JB1 and vehicle in the later phase in LPS-induced models. After anaesthetizing with sevoflurane, we resuspended the neutrophils in 100 µL of saline. One hour later, alveolar cells were collected by BAL and analyzed by FACS (Figure 7A). M-CSFR^+^Ly6G^-^CD11c^+^CD11b^+^SiglecF^+^ AMs were primarily responsible for efferocytosis during the recovery phase of LPS-induced lung injury, when extrinsic UV-irradiated PKH26-labelled apoptotic neutrophils were intranasally injected (Figures 7B). The phagocytic rates of PKH26^+^ neutrophils by AMs were significantly lower in mice receiving JB1 compared to those receiving vehicle, although there were no differences in the percentages and the number of AMs among all monocyte lineage cells (Figures 7C-E).

### In vitro phagocytosis dynamics in MDMs under IGF-1/IGF-1R and LPS stimulation

**Figure 8.**
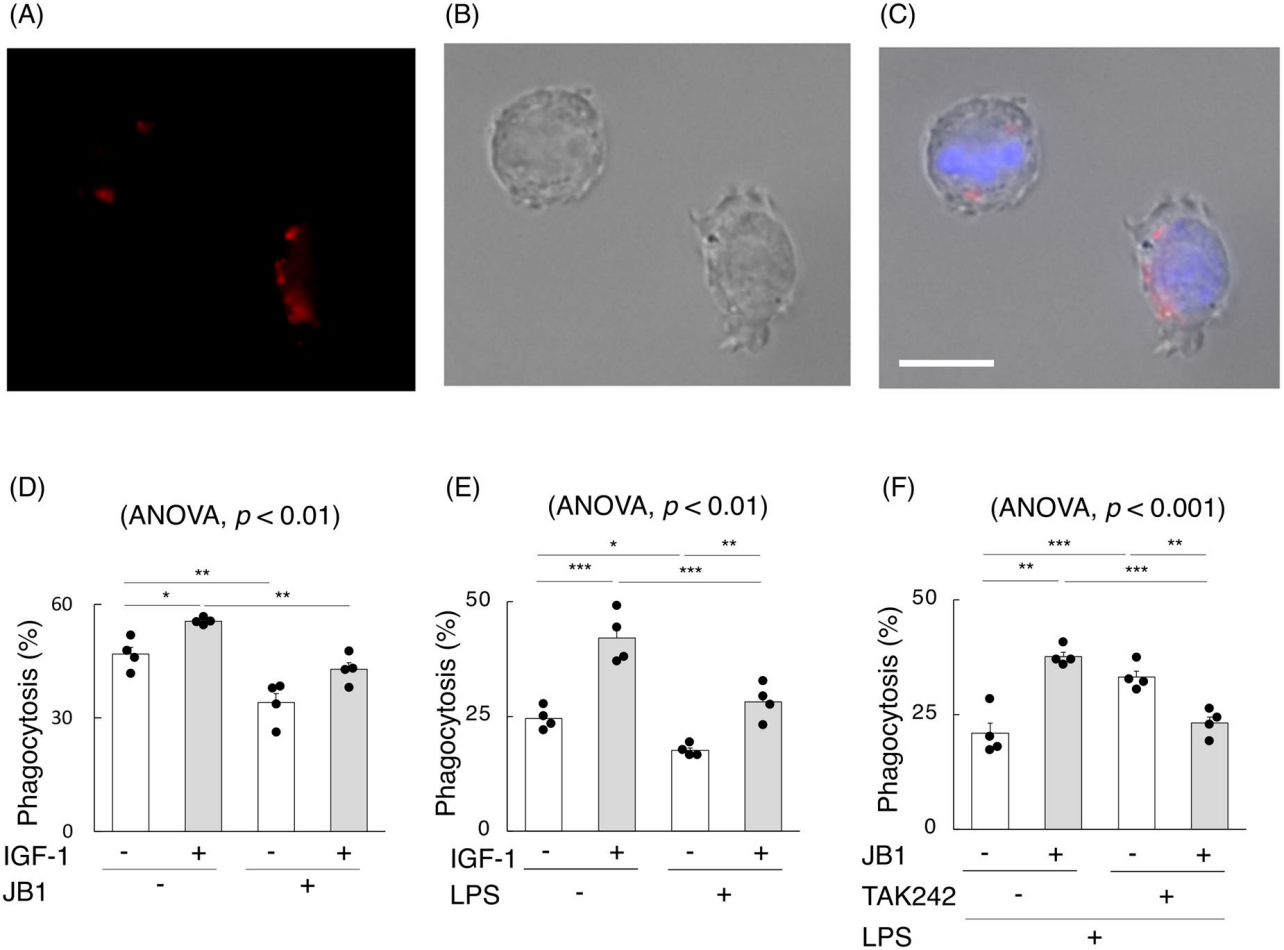
Impact of LPS on IGF-1 and JB1 in an *in vitro* phagocytosis assay. (A–C) Representative immunofluorescence images of bone marrow-derived macrophages and pHrodo-labelled neutrophils (*red*) in phagocytosis chamber slide assays. Nuclei were labelled with 4’6-diamidino-2-phenylindole (*blue*). Scale bar, 10 µm. (D-F) In phagocytosis chamber slide assays using bone marrow-derived macrophages (MDMs) obtained from C57BL/6J mice. (D) The enhancing effects of recombinant insulin-like growth factor-1 (IGF-1) were evaluated with or without JB-1 (n = 4 samples per group). (E) The enhancing effects of recombinant IGF-1 with or without lipopolysaccharide (LPS) were estimated (n = 4 samples per group). (F) The effects of JB1 and TAK242, an antagonist of Toll-like receptor-4, were estimated under LPS stimulation (n = 4 samples per group). The data are presented as bar graphs and dot plots showing the mean ± standard error of the mean (SEM). Differences among multiple groups were analyzed using two-way ANOVA followed by Tukey’s *post hoc* tests. *p < 0.05, **p < 0.01, ***p < 0.001.

A phagocytosis chamber slide assay was performed using MDMs from C57BL/6J mice (Figure 8A–C). The proportion of cells showing digestion of pHrodo-labelled neutrophils significantly increased upon treatment with recombinant IGF-1 (Figure 8D). JB1 decreased uptake rates in MDMs more than those cocultured with vehicle. Efferocytosis capacity of MDMs cocultured with recombinant IGF-1 and JB1 significantly decreased compared to those cocultured with recombinant IGF-1 and vehicle. No difference was observed in neutrophil uptake counts by MDMs cocultured with JB1, regardless of the presence or absence of recombinant IGF-1 (Figure 8D). Furthermore, although LPS inhibited the phagocytic rates in MDMs, recombinant IGF-1 improved the phagocytic rates in MDMs cocultured with LPS (Figure 8E). Conversely, under LPS stimulation, the phagocytic rate of MDMs cocultured with JB1 was significantly increased compared to those cocultured with the vehicle. However, when TAK242, an antagonist specific to TRL4 that recognizes LPS as a ligand, was added under LPS stimulation, the phagocytic rates of MDMs cocultured with JB1 were decreased compared to those with vehicles (Figure 8F). Thus, IGF-1 increases efferocytosis capacity regardless of the presence of LPS, whereas JB1 has the opposite effect depending on the presence of LPS.

## Discussion

Numerous reports have examined the therapeutic effects of IGF-1 delivery or IGF-1R inhibition in lung injury models induced by bleomycin, LPS, and hyperoxic conditions [11,14,16,17,19]. Anti-IGF-1 receptor-blocking antibodies and IGF-1 antagonist peptides improve acute inflammation and fibrosis when administered concurrently with the inducers of lung injury [11,14,16]. In contrast, recombinant IGF-1 or IGF-1 protein delivery using lentivirus vectors has been shown to improve lung injury when administered intraperitoneally or intramuscularly concurrently or before these inducers [17,19]. It is not clear why these contradictory results were obtained. The present study demonstrated that recombinant IGF-1 administered during the later phase of LPS-induced lung injury improved the condition of lung injury, while simultaneous administration of JB1 had the opposite effect and worsened the condition. This indicates that the effect of IGF-1 in improving lung injury is mediated *via* IGF-1R signalling, and that IGF-1/IGF-1R signalling may act in different directions between the acute and recovery phases of lung injury.

Prolonged mechanical ventilation in ARDS worsens prognosis, and impairment of macrophage efferocytosis has increasingly been highlighted as a critical issue [3,7–9]. This study was conducted to explore potential solutions to this issue. Intratracheal infusion of MDMs cocultured with IGF-1 improved BAL neutrophil counts in lung injury, while JB1 offset the effects of IGF-1. In addition, the number of lung macrophages was not significantly altered after intratracheal infusion of MDMs cocultured with IGF-1. This suggests that the effect of IGF-1 on lung injury derives from macrophage function itself. Furthermore, IGF-1/IGF-1R signalling increased efferocytosis capacity, consistent with previous findings [15]. We also found that JB1 consistently inhibited the effect of macrophage uptake during the recovery phase of lung injury. This paper is the first to show that IGF1/IGF-1R signalling in macrophage contributes to recovery during the later phase in a lung injury mouse model *via* enhancing effecocytosis capacity.

LPS has been shown to inhibit efferocytosis by MDMs, consistent with previous reports [22]. Interestingly, while IGF-1 improved the efferocytosis capacity of MDM with or without LPS coculture, JB1 promoted efferocytosis by MDMs under LPS stimulation. In contrast, JB1 reduced the efferocytosis capacity of MDMs, when they were cocultured with LPS and TAK242, or in a LPS-free condition. Pathogen-associated molecular patterns, such as LPS, are abundant in the lungs during the acute phase, and are generally expected to be cleared in the recovery phase. The results of the *in vitro* experiments in this study correspond with the findings from the recovery phase in the mouse model where IGF-1R was inhibited, as well as with the results from previously published literature regarding the acute phase in mouse models where IGF-1 inhibition suppressed inflammation [11,14,16]. Thus, IGF-1R signalling may play different phase-dependent roles in acute lung injury models, whereas IGF-1 may consistently ameliorate these conditions regardless of the phases. Our experimental results focusing on the efferocytosis capacity of macrophages may contribute to elucidating the mechanisms behind the previously contradictory results observed with IGF-1 stimulation and IGF-1R inhibition during the acute phase of lung injury. However, the pathways by which IGF-1 signalling facilitates efferocytosis in MDMs under LPS stimulation remain to be fully elucidated.

Another important indicator of recovery from acute lung injury is the clearance of exudates in the alveoli, which is caused by increased alveolar permeability during the acute phase. In this study, IGF-1 administered *via* the airways during the recovery phase did not show a statistically significant reduction in BAL protein levels. Therefore, further investigations measuring the concentrations of various inflammatory mediators are needed to explore effective methods.

Notably, although inhibition of IGF-1/IGF-1R signalling has different effects in a phase-dependent manner during the course of acute lung injury, IGF-1 stimulation consistently improves the conditions from the acute phase to the recovery phase. Lower circulating IGF-1 levels have been reported to be associated with the poor prognosis in ARDS [23]. In conclusion, we highlight that IGF-1 delivery may offer a potential treatment strategy for ARDS in humans.

## Data Availability

The data is available for reproduction of results on request from the corresponding author.
